# Notes of Interesting Obstetrical Cases, with Remarks

**Published:** 1870-11

**Authors:** J. F. Miner


BUFFALO

### Medical and Surgical Journal.

VOL. X. NOVEMBER, 1870. No. 4.

### Original Communications.

ART. I.—*Notes of interesting Obstetrical Cases, with Remarks.* By J. F. MINER, M. D.

CASE 1ST—Mrs. Mc M., aged 26, Primapara. Attended by Dr. Ayer, in natural labour. Child delivered at four o'clock: no symptoms of disease manifest, and the patient left in every respect comfortable. Called again about two hours later, and found patient in convulsions. These continuing, or appearing at near intervals, I was, at Dr. Ayer's request, invited to visit with him. Patient insensible with persistent convulsion; urine about normal in quantity, but albuminous in unusual degree; extremities anasarcous to moderate extent. Dr. A. prescribed Croton oil, gtt. ij. every hour, until free operation. Patient died comatose six hours later.

CASE 2ND—Mrs. R., aged 28, Primapara. Attended by Dr. D· in natural labor, which terminated rapidly. But little anasarca, and no other symptoms of disease. Seized in convulsion about four hours after delivery, which terminated in profound coma and death in six hours. Attempts were made to obtain hydragogue catharsis, but to no effect; coma was too profound to allow the satisfactory administration of medicine. Urine three-fourths albumen

CASE 3RD—Mrs. ——. Attended by an irregular practitioner. Labor said to have been normal; had complained of not being well, and entertained the conviction that she should not survive her confinement. Was visited by her medical attendant after convulsion was present, and received from him powders of sugar and morphine.

After his dismissal I was called to prescribe. Learned that the convulsion appeared three or four hours after termination of labor; seemed comfortable and cheerful, and made no complaint until a few minutes before she was seized with convulsions, when she complained of severe pain in the head. Anasarca well marked, feet very much distended. Bowels had moved before labor; no urine had been known to pass after completion of labor; introduced catheter, but obtained no urine—not a single drop. Attempted to obtain hydragogue catharsis, but without effect. Patient died in about six hours after commencement of convulsion.

It will be observed that my notes of these cases are incomplete; this grows out of the circumstances of observation. In no case was the physician aware of the presence of any symptoms of derangement or disease; was called simply to attend in labor, and wholly unaware of danger. The examination of urine was also imperfect, as no time or opportunity offered for careful chemical or microscopic observation. These three cases came under observation during the last month,* and were all in the same neighborhood, almost within the same square; and to this list I propose to add another, which resembles them in some respects, but which I regard as entirely unlike, yet also occurring in the same vicinity. Uræmic poisoning is perhaps more frequent than is generally supposed, and is associated with pregnancy in a manner which is but imperfectly understood. It seems to me, judging from my own experience alone, that such disease is vastly more common than formerly, but there are so many sources of error in this, that my observation is of no real value. The best statistics now show its presence in the ratio of one to twenty-three, appearing more frequently in primaparæ.

These cases were not observed by any physician until the very termination, and consequently the report is quite unsatisfactory. They are published more for warning than instruction.

---

* May, 1870.

It seems certain that pregnancy is an excitor of albuminuria in persons previously healthy, and that it aids in developing those morbid conditions, when otherwise they might have remained latent for a long period, or might have wholly disappeared.

The presence of albumen in the urine of pregnant women is of uncertain import, since not only may it be present, but casts such as are recognized in Bright's disease, even in advanced stage, may also be plainly observed, and after labor these conditions may entirely disappear. This appears to be fully sustained by the most careful observation. Notwithstanding this, albumen, when present in the urine, and especially if associated with renal casts, is of grave omen, and sufficient to show the intelligent physician that the danger is imminent. The frequency of this condition in pregnancy, and the obscurity often of its general symptoms, should show how very important it is that the condition of the patient during pregnancy be carefully watched, and especially so in primaparæ; the victims of uraemic poisoning are already much too numerous, since early detection would, in many instances, enable us to avoid a fatal issue.

To the foregoing record of observation, I desire to add another case, and make a remark or two, though I do not think I am recording a case which resembles the first three in any important particular, except the termination.

Mrs. S., aged 35, was delivered in natural and easy labor of a small, delicate child; doubtful whether it had reached full time. Mother and child well; mother very cheerful and happy, complaining of no unpleasant symptoms. Thirty-six hours after delivery she wakened her nurse with a sudden cry of pain in the head; asked that her husband be called, and with great difficulty made known some of her wishes. In a few minutes I was at her bedside, and found complete paralysis of right side. She could still make herself understood a little, but could not articulate words distinctly. Pupil of one eye dilated; all sensation and motion on the affected side lost; was still conscious, and understood what was said to her; deglutition not quite impossible, but very difficult. But a short time elapsed before she was perfectly unconscious, breathing stertorously and sinking rapidly. Died in about four hours after first attack. The bowels had moved freely, and the urine had been copious and easily voided.

The diagnosis, to my mind, was positive, and entirely satisfactory. This patient undoubtedly died from apoplectic effusion, though the case did in some respects resemble uraemic poisoning. Uræmic poisoning does not produce paralysis; can never have so sudden an appearance; is always attended by precursory symptoms. The stertorous breathing was too deep for puerperal eclampsia, unless in the advanced stage, when great congestion, or actual effusion had taken place.

These cases, taken together, constitute a remarkable obstetric record for the time and location; and occurring thus, awakened, in some instances, fear and anxiety in those expecting soon to be in labor. So far as uraemic poisoning is concerned, the fatal issue possibly might have been prevented or delayed by timely interference, but the apoplectic effusion was perhaps an accident, against which we could not guard.

